# A Biomimetic Fish Fin-Like Robot Based on Textile Reinforced Silicone

**DOI:** 10.3390/mi11030298

**Published:** 2020-03-12

**Authors:** Sascha Pfeil, Konrad Katzer, Anas Kanan, Johannes Mersch, Martina Zimmermann, Michael Kaliske, Gerald Gerlach

**Affiliations:** 1 Institute of Solid State Electronics, Faculty of Electrical and Computer Engineering, Technische Universität Dresden, 01069 Dresden, Germany; 2 Fraunhofer Institute for Material and Beam Technology IWS, 01277 Dresden, Germany; 3 Institute for Material Science, Faculty of Mechanical Science and Engineering, Technische Universität Dresden, 01069 Dresden, Germany; 4 Institute for Structural Analysis, Faculty of Civil Engineering, Technische Universität Dresden, 01069 Dresden, Germany; 5 Institute of Textile Machinery and High Performance Material Technology, Faculty of Mechanical Science and Engineering, Technische Universität Dresden, 01069 Dresden, Germany

**Keywords:** biomimetics, dielectric elastomer actuators, textile reinforcement, soft robotics, fish fin robot, textile-elastomer compounds, bending structures

## Abstract

The concept of merging pre-processed textile materials with tailored mechanical properties into soft matrices is so far rarely used in the field of soft robotics. The herein presented work takes the advantages of textile materials in elastomer matrices to another level by integrating a material with highly anisotropic bending properties. A pre-fabricated textile material consisting of oriented carbon fibers is used as a stiff component to precisely control the mechanical behavior of the robotic setup. The presented robotic concept uses a multi-layer stack for the robot’s body and dielectric elastomer actuators (DEAs) on both outer sides of it. The bending motion of the whole structure results from the combination of its mechanically adjusted properties and the force generation of the DEAs. We present an antagonistic switching setup for the DEAs that leads to deflections to both sides of the robot, following a biomimetic principle. To investigate the bending behavior of the robot, we show a simulation model utilizing electromechanical coupling to estimate the quasi-static deflection of the structure. Based on this model, a statement about the bending behavior of the structure in general is made, leading to an expected maximum deflection of 10 mm at the end of the fin for a static activation. Furthermore, we present an electromechanical network model to evaluate the frequency dependent behavior of the robot’s movement, predicting a resonance frequency of 6.385 Hz for the dynamic switching case. Both models in combination lead to a prediction about the acting behavior of the robot. These theoretical predictions are underpinned by dynamic performance measurements in air for different switching frequencies of the DEAs, leading to a maximum deflection of 9.3 mm located at the end of the actuators. The herein presented work places special focus on the mechanical resonance frequency of the robotic setup with regard to maximum deflections.

## 1. Introduction

Electroactive polymers (EAPs) are some of the most promising materials when it comes to new propulsion mechanisms in the field of soft robotics. In comparison to the well-established rigid robotic concepts, soft robots inherit the ability to implement core-functions like sensing, actuation, or computing on the level of material properties [1]. The class of dielectric elastomers (DEs) and, thus, DEAs offer great potential to fulfill several main functions of such soft robots. Especially, the dielectric elastomer actuators (DEAs) show highly promising possibilities with regard to applications in the field of soft actuation [2]. They consist of a thin elastomer membrane sandwiched between two compliant electrodes [3]. At an applied voltage on the electrodes, the charge separation leads to an electric field between the electrodes. Due to the quasi incompressibility, the dielectric elastomer material is forced by the electrostatic pressure to expand in all free spatial directions. The resulting expansions can reach strain levels of more than 300% [4].

The rather simple concept of a plate capacitor can also be used for sensing applications where a deformation leads to measureable changes of capacitance [5], voltage [6], or resistance [7]. Such sensor concepts are essential for soft robotics since they allow integrated sensing to merge actuator and sensor functionalities [8]. Together with DE-based generators for energy harvesting [9], dielectric switches [10], and logic gates based on these switches [11], prospective self-sustainable robots can be built [12].

In 2016, the first fully functional soft robot, the octobot, was presented [13]. The octobot is an example of some general trends in soft robotics. Its versatility allows using it in different surroundings with different actuation modes. Together with its simplicity in design and material choice, the entire robot fits perfectly into biomimetic applications as considered in this work.

According to Vincent et al. [14], biomimetics is a research field that embraces “the practical use of mechanisms and functions of biological science in engineering, design, chemistry [and] electronics”. Biomimetics take their inspiration from nature and mimic naturally acting mechanisms and concepts. In the field of soft robotics, there are different approaches to the idea of biomimetics. One major issue is biocompatibility, which can be achieved due to material choice wherever possible. Many soft robotic concepts use silicones as the base material [15], which shows at least mechanical biocompatibility and, in the case of modified silicones, even full biocompatibility [16]. Besides the material properties, the actuation itself is in the focus of biomimetic soft robots. The common ground lies in adapting natural forms of movement. There are various concepts based on nature inspired movements such as worm-like robots [17], walking structures [18], grippers [19], or fins [20].

Here, we present a fish fin-like biomimetic soft robot. While textile materials in soft robotics are so far mostly used to modify the surfaces of robots [21], the presented approach uses a pre-fabricated textile material to enhance the mechanical properties of a soft structure. The textile material’s purpose is to both stabilize the structure and to manipulate its mechanical bending properties. Together with the silicone matrix cast around it, it gives the typical fish fin shape to the robot. DEAs on both outer sides of the robot initiate the bending movement, which can lead to a propulsion. Similar to other approaches for fish-like robots [22,23,24], the herein presented robot uses body propulsion, generating an undulating wave towards the end of the fin as the propulsion method. This means that the actuators are located on the body part of the robot, and the fin part at the end of the robot is passively excited by the generated bending movement at the front parts. The design of the robot follows the approach of generating a maximum deflection at the end of the fin.

The main contribution of this work lies in the use of pre-fabricated textile materials as the reinforcement structure. While other work presented similar fish fin-like concepts, none of them have used a pre-fabricated and tunable textile material so far. The textile material allows the tuning of the mechanical properties for the whole robot while ensuring lightweight properties. The mechanical properties are pre-defined due to the processing of the textile fabric, which lowers the induced deviations at the processing and thus improves reproducibility.

Different from other DEA-driven fish robots [24], the presented work uses equi-biaxial pre-stretched membranes for the DEAs, allowing thinner resulting dielectric membranes, which makes the robot more efficient with regard to the generated forces and operating voltages. Due to their balanced pre-stretch ratio, the DEAs hold the robot in a straight aligned position in its initial state. The presented work also uses a method for minimal wiring for the underwater actuation by utilizing the water as the common ground electrode. In this work, we present the theoretical background for the bending motion induced by the DEAs, together with a finite element simulation of the electromechanical coupled deformation. We describe the manufacturing and the characterization of the mechanical performance of the robot to find a maximum deflection depending on the switching frequency of the DEAs. In addition, a first impression of the possible versatility of the robot with regard to its use in water and in air is given. Possible applications under water with minimum electrical wiring are proven with regard to possible autonomous actuation in future stages of development.

## 2. Materials and Methods

### 2.1. Robotic Concept

**Figure 1:**
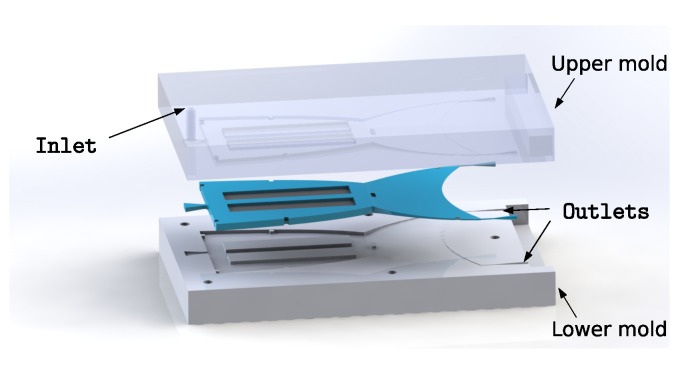
Cast mold for the robot’s body. The two halves form the cavity for the textile-elastomer compound inside. The silicone mixture is pressed through the cast mold, injected on the inlet, and overfilled towards the outlet.

The robot was designed in a way to mimic the typical waving motion of a fish fin. According to [25], the robotic concept used body propulsion by bending the fish’s body into a backward-moving wave that traveled towards the end of the fin. The geometry itself was inspired by emarginate fin shapes, providing a good tradeoff between low drag due to the low surface area of the fin and good acceleration force. The implemented setup consisted of multiple layers of different materials. In the neutral plane in the middle of the setup, a highly anisotropic textile material was placed. It was pre-fabricated in a separate step and consisted of a carbon fiber unidirectional (CF-UD) tape that was made from Sigrafil C T50-4.4/255-E100 fibers (SGL Carbon SE, Wiesbaden, Germany) with a grammage of 200 $\frac{g}{m^{2}}$ [26]. To ensure good adhesion of the matrix material to the fibers and maximum infiltration, they were coated with a low-viscosity styrene butadiene rubber (SBR) system using a Basecoater BC 32 (Coatema GmbH, Dormagen, Germany) roll-to-roll machine. The coating material was crosslinked at 160 °C, as recommended. To get the fabricated textile material in the desired shape of the fish fin, the CF-UD-tape was cut using a solid-state laser with a power of 5 kW and a wavelength of 1.064 μm. The textile material conditioned in this way was then placed in a cast mold to infiltrate it with a silicone mixture, Sylgard 184 (Dow Silicones, Wiesbaden, Germany). The cast mold used was 3D-printed using an acrylonitrile butadiene styrene (ABS) copolymer filament that was smoothed after printing (Figure 1).

**Figure 2:**
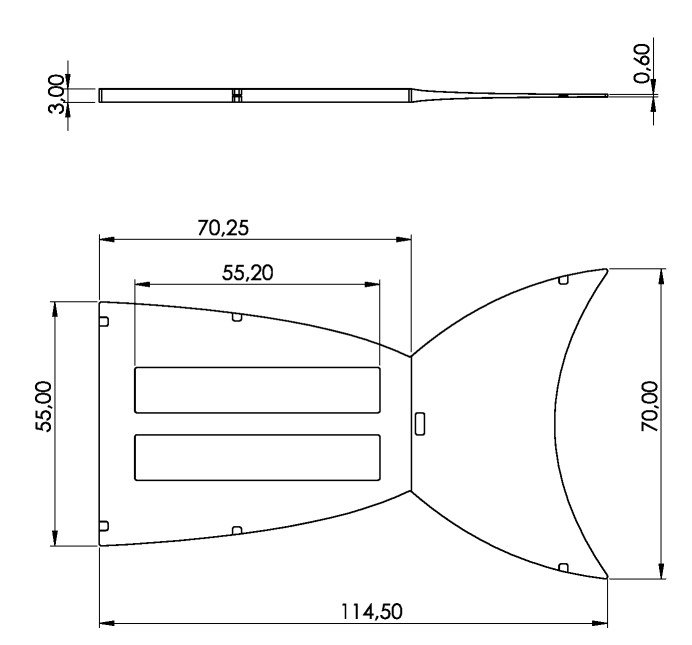
Dimensions of the fish fin structure as the core part of the robot’s body in mm. Rectangle parts represent void spaces down to the neutral plane, holding the textile material.

The geometry of the cast mold defined the design of the robot’s body. The core part of the robot was designed with void spaces in its structure that reached down to the neutral plane of the robot. These parts formed spacers in the cast mold that held the uninfiltrated textile material in its position inside the mold. The central gaps on the robot’s body were later covered by the DEAs. To produce the composite material for the fish’s body, the prepared textile material was placed in the mold and fixed in position by locking the mold halves. The prepared cast mold was filled with the silicone mixture and afterwards put into an oven at 60 °C to cure the silicone mixture within 24 h. After releasing the cured textile-elastomer compound, it could be assembled with the DEAs. Figure 2 shows the dimensions of the robot’s body after curing.

The actuators were prepared in another separate step. For that, a 100 μm Elastosil 2030 (Wacker, Munich, Germany) silicone foil was equi-biaxially pre-stretched to 140% and covered with electrodes. The pre-stretching of the silicone foil offered multiple advantages for use in dielectric actuator applications [27,28]:

- It increased the electrical breakdown-strength of the material.
- The pull-in instability was suppressed.
- The silicone membrane was kept flat and defined in area, leading to a defined actuation.
- The operating point for the actuation was set in a beneficial region of the hyperelastic stress-strain curve of the dielectric material.

**Figure 3:**
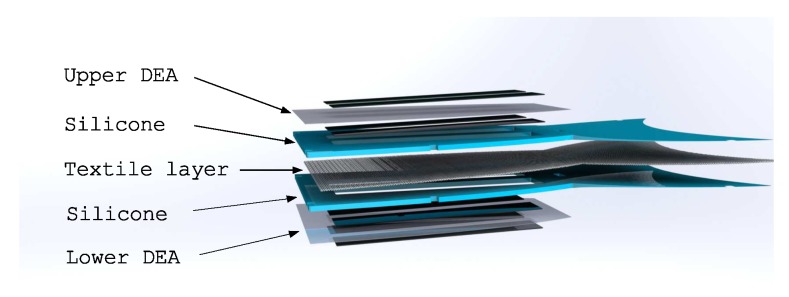
Layered setup of the robot with DEAs on both outer sides and the textile layer in the neutral plane sandwiched between silicone layers.

The electrodes for the actuators were applied on the pre-stretched dielectric membrane, using an air-brush method, spraying a mixture of room temperature curing silicone Ecoflex 00-10 (KauPo, Spaichingen, Germany), heptane, and carbon black. The electrode areas were defined using masks in the shape of the desired geometry to cover the corresponding regions of the silicone foil. The electrode mixture was then sprayed on the masks and afterwards cured at 60 °C for another 8 h to form a stable bond to the Elastosil foil. The prepared DEAs were glued on both outer sides of the robot’s body (Figure 3).

**Figure 4:**
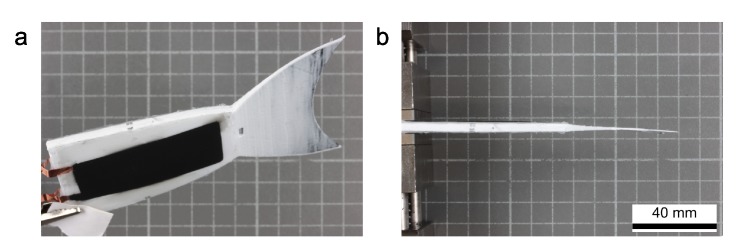
Assembled robotic setup. (**a**) General view with DEAs and electrical wiring and (**b**) top-view to illustrate the straight alignment due to the balanced pre-stretch ratios of the two DEAs.

As mentioned before, the DEAs were made of 100 μm thin silicone films. The textile layer had a thickness of 0.4 mm between two layers of 1.3 mm silicone material. The electrical contacting was realized with copper wires that were glued to the corresponding positions on the robot. Figure 4 shows a photograph of the complete robotic setup.

### 2.2. Bending Movement and Force Generation

**Figure 5:**
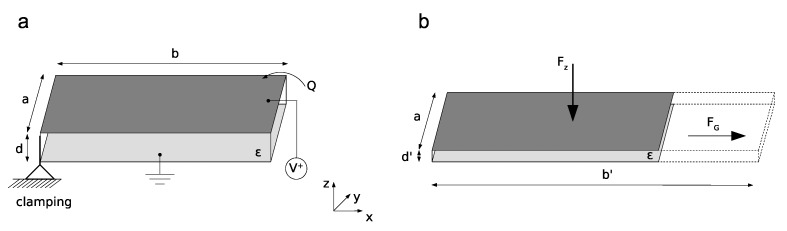
Actuator geometry based on a plate capacitor. (**a**) Initial undeformed state, clamped at the front, and (**b**) deformed state with the generated forces leading to an elongation of the actuator. The boundary conditions limit the deformation in the *y*-direction.

The bending movement resulted from the force generated by the DEAs on both outer sides of the robot. The robot itself was designed in such a way that it allowed bending only in the longitudinal direction since the textile material limited an expansion in the transversal direction. The generated force of the DEAs drove the bending movement. The DEAs could be assumed as a plate capacitor with the reference potential at the ground electrode on the lower plate (Figure 5).

The force considered here described the force of the upper electrode acting towards the lower electrode. To derive the Maxwell pressure generated between the electrodes, we used the Maxwell stress tensor approach based on electromechanical coupling of the DEA. According to [29,30], the Maxwell stress tensor can be written as:

(1) $$\vec{T}=\vec{E}⊗\vec{E}+\vec{B}⊗\vec{B}-\frac{1}{2}\cdot \epsilon_{0}\left(E^{2}+B^{2}\right),$$

which is equivalent to:

(2) $$\vec{T}=\epsilon (\vec{E}\cdot \vec{E^{T}}-\frac{1}{2}\cdot I\cdot {\left|E\right|}^{2})+\frac{1}{\mu }(\vec{B}\cdot \vec{B^{T}}-\frac{1}{2}\cdot I\cdot {\left|B\right|}^{2}),$$

with the electrical field strength $\vec{E}$, the dielectric permittivity ϵ, the magnetic field strength $\vec{B}$, the magnetic permeability μ, and the unit matrix I. Due to the quasi electrostatic case, we could assume the magnetic field strength as zero, which simplifies Equation (2) to:

(3) $$\vec{T}=\epsilon (\vec{E}\cdot \vec{E^{T}}-\frac{1}{2}\cdot I\cdot {\left|E\right|}^{2}).$$

The first step towards an expression for the Maxwell stress is to describe the electric field *E*, caused by the charge distribution $\sigma_{A}$ on the upper electrode, which contains the charge Q over the area *A* of the electrode:

(4) $$\vec{E}=\frac{Q}{\epsilon A}=\frac{\sigma_{A}}{\epsilon }\cdot {\vec{e}}_{x,y,z}.$$

The derived equation for the electric field can be inserted into Equation (3):

(5) $$\begin{array}{cc} \vec{T} & =\epsilon [\left(\begin{array}{c} E_{x}\\ E_{y}\\ E_{z} \end{array}\right)\cdot \left(E_{x};E_{y};E_{z}\right)-\frac{1}{2}\left(\begin{array}{ccc} 1 & 0 & 0\\ 0 & 1 & 0\\ 0 & 0 & 1 \end{array}\right)\cdot E^{2}]\\ \; & =\frac{\epsilon }{2}\left(\begin{array}{ccc} E^{2} & 0 & 0\\ 0 & -E^{2} & 0\\ 0 & 0 & -E^{2} \end{array}\right). \end{array}$$

Here, $E_{x}$, $E_{y}$, and $E_{z}$ are the components of the electric field in the *x*-, *y*-, and *z*-direction, and *E* is the amount of the electrical field in all spatial directions. The force impact on the planar electrode can be expressed by:

(6) $$\vec{F}={\;\int \int \;}_{A}\vec{T}\cdot d\vec{A}.$$

The differential area element $d\vec{A}$ is given as:

(7) $$d\vec{A}=dxdy\cdot {\vec{e}}_{z}$$

in Cartesian coordinates, where ${\vec{e}}_{z}$ is the unit vector in the *z*-direction. The force component $F_{z}$ in the *z*-direction thus results in:

(8) $${\vec{F}}_{z}={\;\int \int \;}_{A}\vec{T}\cdot {\vec{e}}_{z}={\;\int \int \;}_{A}-E^{2}=-\frac{\epsilon \cdot \sigma_{A}^{2}}{2\epsilon^{2}}\int_{0}^{b}\int_{0}^{a}dxdy.$$

Solving the integral, the final expression for the force impact on the planar electrode is:

(9) $$F_{z}=-\frac{\sigma_{A}^{2}}{2\epsilon }\cdot A.$$

In order to derive an equation consisting of practically useable quantities, the expression of the force can be reformulated by using the applied voltage *V* instead of the charge distribution $\sigma_{A}$. For that, the electric potential $\Phi_{o}$ of the upper electrode can be expressed as:

(10) $$\Phi_{o}=\Phi_{i}-\int_{0}^{d}E\left(z\right)dz,$$

where $\Phi_{i}$ is the potential on the lower electrode. Inserting Equation (4) into Equation (10) and solving the integral leads to:

(11) $$\Phi_{o}=\Phi_{i}-\frac{\sigma_{A}}{\epsilon }\cdot d.$$

Introducing the applied voltage *V* as the difference of the both electric potentials yields:

(12) $$V=\Phi_{o}-\Phi_{i}=-\frac{\sigma_{A}}{\epsilon }\cdot d.$$

Using this formulation, the charge distribution $\sigma_{A}$ can be described as:

(13) $$\sigma_{A}=-\frac{V\cdot \epsilon }{d}.$$

The resulting force can be derived by inserting Equation (13) into Equation (9):

(14) $$F_{z}=-\frac{\epsilon^{2}V^{2}A}{2d^{2}\epsilon }.$$

This leads directly to the stress component $\sigma_{\mathrm{maxwell}}$ in thickness direction z due to the electric voltage between the electrodes:

(15) $$\sigma_{\mathrm{maxwell}}=\frac{F_{z}}{A}=-\frac{\epsilon V^{2}}{2d^{2}}.$$

Due to the almost incompressible silicone material, it can be assumed that this stress also acts on the cross-sectional area $A_{cross}$ of the electrodes to generate a longitudinal expansion of the DEAs. The initiated force $F_{G}$ can be described as:

(16) $$F_{G}=\sigma_{\mathrm{maxwell}}\cdot A_{\mathrm{cross}}=\frac{\epsilon V^{2}}{2d^{2}}\cdot d^{\prime }a.$$

The membrane thickness $d^{\prime }$ of =51μm in the pre-stretched state and the width of the electrodes *a* of 25 mm give the cross-sectional area $A_{\mathrm{cross}}$ of the DEAs of 1.275 mm^2^. Assuming a dielectric permittivity ϵ of $2.8*0.8854*10^{-}11\frac{A\cdot s}{V\cdot m}$, the generated force for an applied voltage of 3000 V equals 0.055 N and 0.097 N for a voltage of 4000 V. This expanding force leads to the bending movement of the robot. By switching the voltage at the DEAs, a change of the bending direction is initiated. The consecutively switching then generates the waving motion of the robot.

### 2.3. Simulation of the Bending Structure

In order to verify that the desired mechanical behavior of the bending structure can be achieved, a tailored simulation model was developed and utilized. The model is capable of describing the electromechanical coupling of the DEA material. To simplify the relatively complex structural setup, the following assumptions were made:

- The structure was composed of a fiber-reinforced passive material with internal cavities and active DEAs.
- It had a nonlinear geometry, and its bending deformation was based on a coupled electromechanical response.
- Therefore, a nonlinear material model suitable for the simulation of large deformations was used along with an electromechanical finite element.

In the first step, finite element analyses were used to simulate tensile stresses within both the passive and the DEA materials in order to identify the mechanical material parameters. Afterwards, the electromechanical response and the bending deformation of the whole fish fin structure were modeled and demonstrated. The used material model was based on a free energy density function with an additive form as:

(17) $$\Psi^{\mathrm{tot}}=\Psi^{\mathrm{iso}}+\Psi^{\mathrm{ani}}+\Psi^{\mathrm{coup}},$$

where $\Psi^{\mathrm{iso}}$ is an isotropic mechanical contribution, $\Psi^{\mathrm{ani}}$ expresses the anisotropic response due to fiber reinforcement, and $\Psi^{\mathrm{coup}}$ describes the coupled electromechanical behavior. The simulation of the isotropic hyperelastic response was based on the extended tube model [31,32], which was capable of realistically predicting the response of hyperelastic rubber-like materials. The anisotropy due to fiber reinforcement was modeled as was proposed in [33]. Furthermore, the coupled electromechanical energy contribution is defined as:

(18) $$\Psi^{\mathrm{coup}}=-\frac{1}{2}\epsilon \;{\vec{E}}_{L}\cdot C^{-1}\cdot {\vec{E}}_{L},$$

with the electric permittivity ϵ of the material, the electric field vector in the undeformed configuration ${\vec{E}}_{L}$, and the right Cauchy–Green tensor C. The coupled energy function defined by Equation (17) renders a Maxwell stress tensor $\sigma_{\mathrm{maxwell}}^{\prime }$ for the three-dimensional case, which in the case of planar geometries, can be reduced to the simplified Maxwell stress $\sigma_{\mathrm{maxwell}}$ as follows:

(19) $$\sigma_{\mathrm{maxwell}}=\epsilon \;{\left(\frac{V}{d}\right)}^{2}=\epsilon \;E^{2},$$

with the deformed thickness *d* of the planar dielectric, the voltage difference *V* between the electrodes, and the one-dimensional electric field *E*. However, due to the fact that the fish fin structure deformed three-dimensionally, we adopted the three-dimensional formulation as given in Equation (18), where the electric field is the vector ${\vec{E}}_{L}$ and the Maxwell stress is the tensor $\vec{T}$. For more details about the continuum theory of electro-elasticity, we refer the reader to [34]. Regarding the numerical implementation, a mixed electromechanical finite element was implemented. Mixed finite elements are needed to overcome the problem of locking in quasi incompressible materials and bending-dominated structures. For more details about quasi incompressible finite element formulations and the numerical modeling of electro-elasticity, see for example [35,36], respectively. The used material model and the coupled finite elements were implemented in an in-house finite element program.

**Figure 6:**
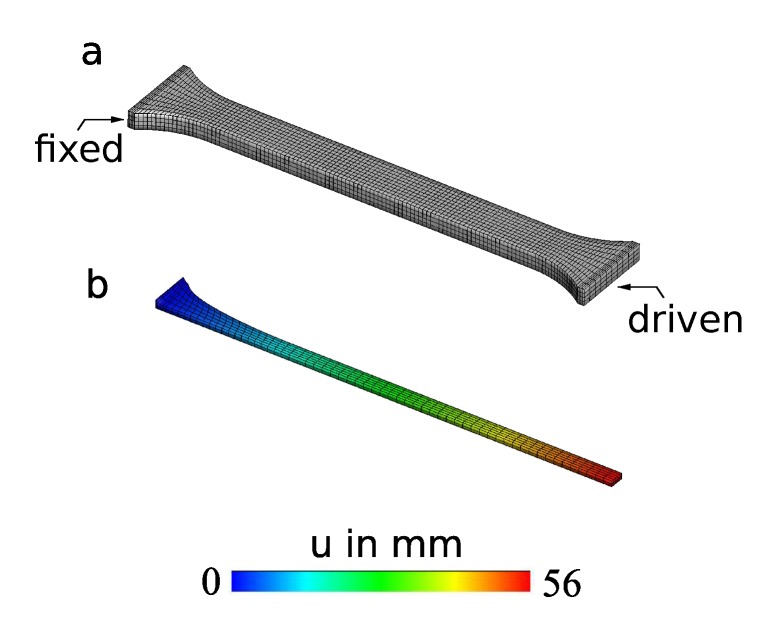
Finite element model for Sylgard 184 silicone elastomer: (**a**) discretization and boundary conditions for a DIN EN ISO 527-2 1A specimen and (**b**) simulation of the tensile test. The contour shows the longitudinal displacement.

The characterization of the used materials was performed by carrying out quasi-static tensile tests for both Sylgard 184 silicone, which was used as the passive matrix of the fish fin, and Elastosil 2030, which was used for the DEAs. These experiments were performed to identify the mechanical material parameters, in order to use them in the electromechanical simulation. The finite element discretization and simulation of a DIN EN ISO 527-2 1A specimen for Sylgard 184 silicone are demonstrated in Figure 6. As symmetric boundary conditions and deformation states could be assumed, the response of only one eighth of the real specimen was simulated as is depicted in Figure 6b. Moreover, a simulation of a simple rectangular specimen for the Elastosil 2030 material was performed, but for the sake of brevity is not shown in this manuscript.

**Figure 7:**
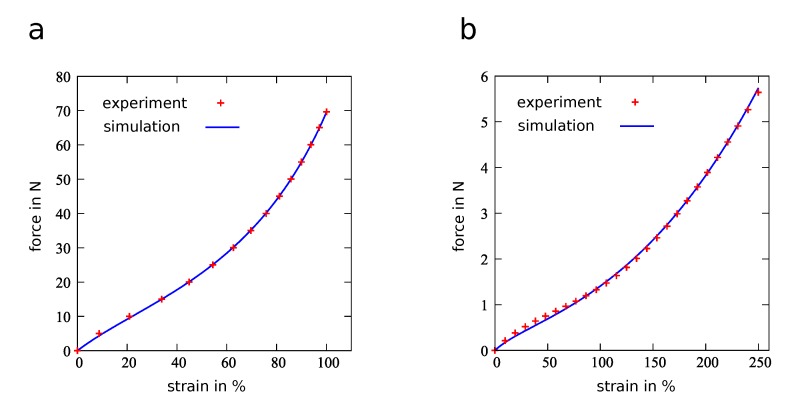
Fitted simulation and experimental results for specimens from (**a**) Sylgard 184 material with DIN EN ISO 527-2 and (**b**) Elastosil 2030 material with a size of 104 mm × 20 mm × 0.1 mm.

Both of the investigated materials showed a hyperelastic force-strain behavior with a higher force response for the Sylgard 184 material compared to the Elastosil 2030 material. The results of the numerical simulations showed good agreement with the experimental data for both specimens as is depicted in Figure 7.

**Figure 8:**
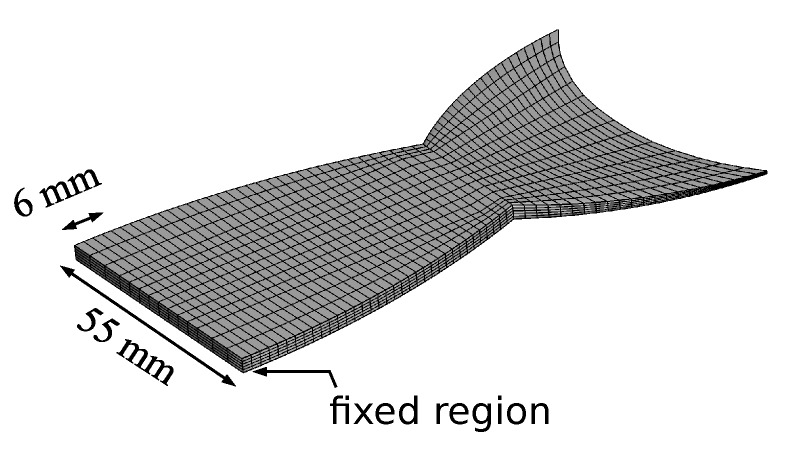
Finite element discretization and mechanical boundary conditions of the fish fin structure. The given values describe the region where mechanical constraints are applied.

The nonlinear geometries of the fish fin matrix and the attached DEAs were taken into account by discretizing them using hexahedral finite elements. Moreover, it was attempted to mimic the real mechanical boundary conditions by applying displacement constraints within the front region as is shown in Figure 8.

**Figure 9:**
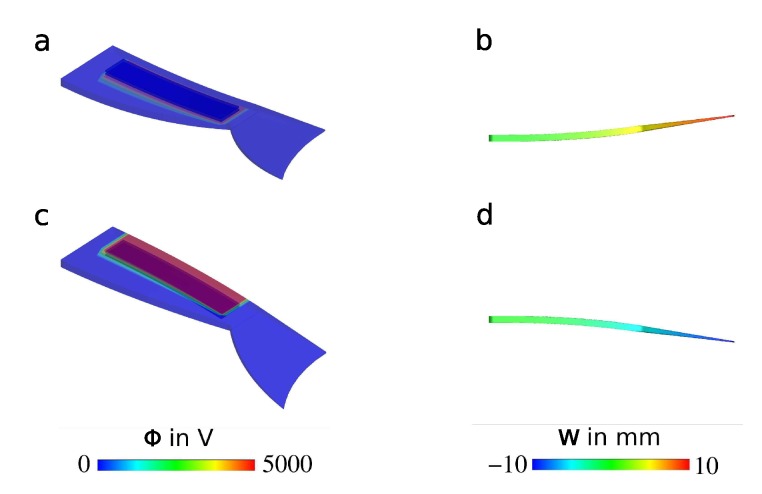
Quasi-static finite element simulation of the fish fin structure from Figure 8 with 3D views (**a**,**c**: voltage scale) and side views (**b**,**d**: deflection scale); the response of the structure due to the stimulation of (**a**,**b**) the bottom DEA and (**c**,**d**) the top DEA.

Regarding the simulation of the coupled electromechanical response, a relative dielectric permittivity $\epsilon_{r}=2.8$ was used as a material parameter. For the mechanical response, the material parameters of the extended tube model were chosen such that the material was considered as a neo-Hookean solid, which was suitable for the realistic modeling of strain values of up to 20%. In this way, some computational costs were saved. This approach was reasonable due to the fact that the fish fin showed large deformations, but did not exhibit large strains. As the boundary conditions were symmetric, only one half of the structure was analyzed. The simulation of the coupled behavior was performed by applying a voltage difference Φ between the two surfaces of the attached DEAs. However, only the rectangular region covered by the electrodes of the DEA was activated. That emulated the real boundary conditions, where rectangular electrodes of limited size covered the surface of the structure. The quasi-static simulation was performed in which a potential difference was increased from 0 V to 5000 V stepwise, and the mechanical response was studied in terms of the displacement *w*. Figure 9 depicts the response of the bending structure upon the activation of the lower and the upper DEA for the final simulation step, where a potential difference of 5000 V was applied.

The results of the quasi-static simulation (Figure 9) showed that a displacement of up to 10 mm at the end of the structure could be expected when the robot was activated with a voltage of 5000 V.

### 2.4. Electromechanical Network Model

**Figure 10:**
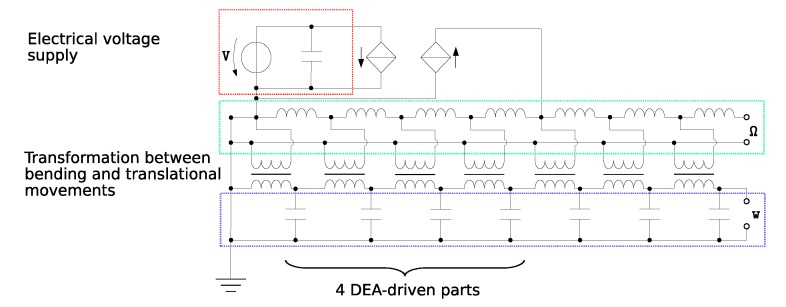
Electromechanical network model of the bending structure, red: electrical domain, green: rotational domain, blue: translational domain.

To estimate the behavior of the complex electromechanical structure of the fish fin, equivalent electromechanical network models were used. Here, the mechanical structure was also modeled as an electrical network while relying on the analogies between the mechanical and the electrical domain. In particular, resonance frequencies could be determined easily. In the case of the fish fin structure, we used the Firestone analogy [37] equating between force and current as the flow quantities and between velocity and voltage as the cross quantities. The procedure allowed coupling the electrical and mechanical domains through transducers, in this case the DEAs, which transduced the applied voltage to a force. This force subsequently moved the fish’s body, which was simplified as a bending beam. The fish fin was discretized into seven sections following the approach for an active bending beam in [38]. Because the description was based on Euler–Bernoulli beam theory, which disregards shear effects and is suited for small deformations, the overall stiffness and consequently the resonance frequency of the beam were overestimated. To implement the described model for the fish fin structure, the simulation tool LTSpice [39] was used. The system parameters were identified by using the CAD data of the robot, textile physical measurements of the reinforcement textile material, and the elastomer’s properties provided by the manufacturer. In the electromechanical network model, the DEA acted on the first four segments of the discretized fish fin and was considered as clamped on the left side, which related to a shorted circuit and a free end on the right side. Figure 10 illustrates the coupling of the electrical (red), rotational (green), and translational (blue) domains in the equivalent network model with the rotational speed Ω and the resulting deflection w.

**Figure 11:**
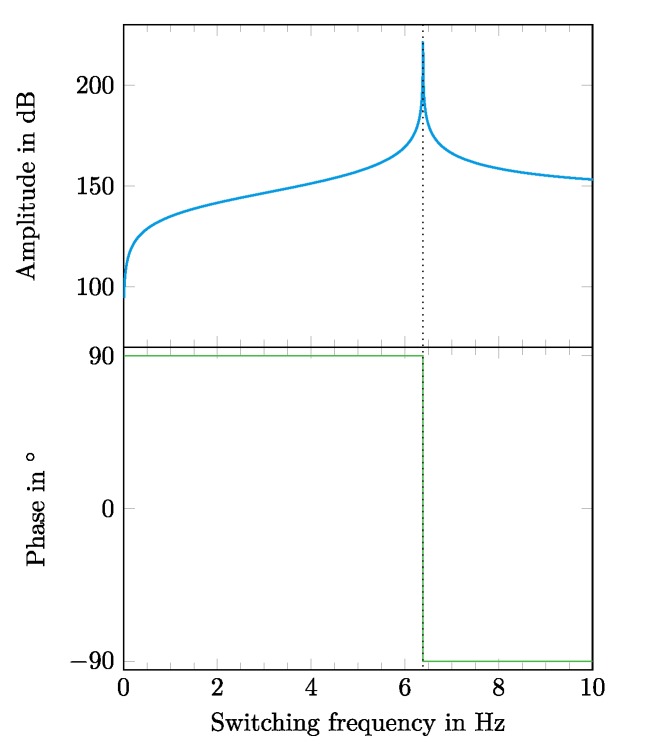
Frequency response of the amplitude and phase of the deflection w at the end of the fish fin from Figure 8 and Figure 10, respectively. The resonance frequency of 6.385 Hz is marked.

The behavior of the bending structure was simulated by changing the frequency input of the network model. The voltage was simulated with a sine-waveform with a frequency in the range between 0 Hz and 10 Hz. Figure 11 shows the frequency response with respect to the amplitude and phase of the deflection w of the modeled structure.

The predicted resonance frequency was located at 6.385 Hz. Since the used model was expected to overestimate the resonance frequency, the experimentally determined one was expected to be lower.

### 2.5. Image Correlated Measurement

**Figure 12:**
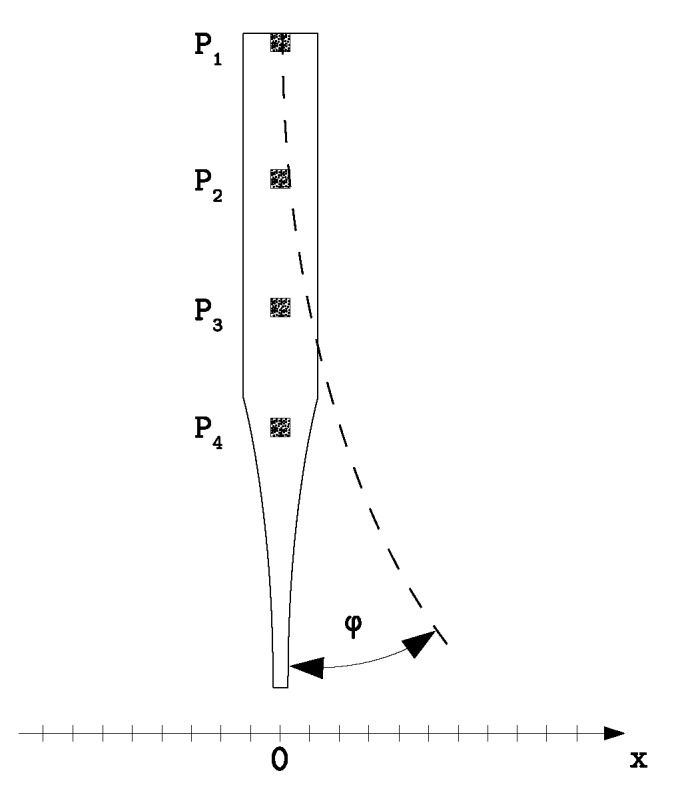
Top-view of the measurement setup with speckle patterns P1–P4 on four positions along the top-side of the robot. The angular displacement φ leads to a deflection of the speckle patterns in the *x*-direction that are captured by the camera.

To evaluate the oscillating motion of the fish fin, a digital image correlation (DIC) measurement system ARAMIS 5M (GOM, Germany) was used to perform camera-based displacement measurements. The used camera had a resolution of 2448 × 2050 pixels and a focal length of 50 mm. A lowering of the resolution to 1224 × 1025 pixels allowed a recording frame rate of 29 Hz. After the calibration with a ceramic calibration target, the system was able to detect length differences of a few micrometers. The subset size was set to 19 pixels and the distance to 15 pixels. To measure the displacements along the bending robot, four printed speckle patches were adhered at different positions on the backbone of the robot. The freeware SpeckleGen (Correlated Solutions, USA) provides a pdf file with customizable speckle patterns. These patterns were printed and then glued onto the fish fin. The positions of the speckle patches were located equi-distant along the first 70 mm, which meant the active part with the DEAs. A schematic of the setup can be seen in Figure 12.

The DEAs were activated with a voltage of 4000 V with a square-waveform at different switching frequencies between 1 Hz and 7 Hz. The camera recorded the robot’s movement and the positions of the speckle patterns in each video frame. For the measurement, the robot was clamped at the front side and held in a hanging position as depicted in Figure 12. The different displacement values were extracted after the recording by analyzing the collected data with the ARAMIS software. For that, the upper speckle pattern was used as the reference pattern since it was clamped and not moving with the bending motion. The deflections in the *x*-direction were then calculated by forming the three displacement differences ${\mathrm{dx}}_{i}$ in relation to the x position of speckle P1,

(20) $$\mathrm{dx}2=x_{P2}-x_{P1},$$

(21) $$\mathrm{dx}3=x_{P3}-x_{P1},$$

(22) $$\mathrm{dx}4=x_{P4}-x_{P1}.$$

## 3. Results and Discussion

### 3.1. Displacement Measurement and Mechanical Resonance

**Figure 13:**
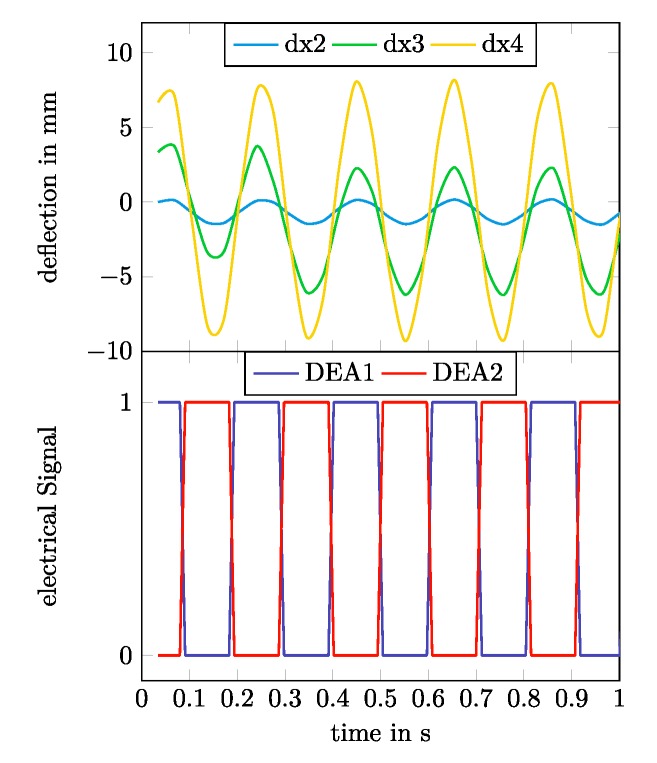
Exemplary deflection results for a switching frequency of 5 Hz. Overview of the electrical switching regime and the caused deflections dx2, dx3, and dx4.

Displacement measurements were performed for different switching frequencies of the DEAs between 1 Hz and 7 Hz with a smaller step width of 0.1 Hz in the range between 4.5 Hz and 5.5 Hz. Figure 13 shows the switching regime of the two DEAs on both outer sides of the robot together with the measured deflection differences ${\mathrm{dx}}_{2}$…${\mathrm{dx}}_{4}$ between the four speckle patterns from Figure 12 for a switching frequency of 5 Hz.

**Figure 14:**
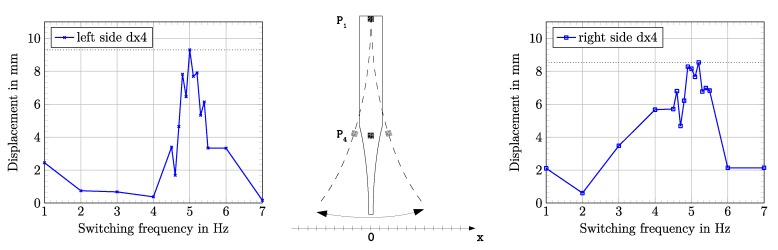
Results for the measured deflections dx4 to the left and to the right side over the investigated frequency range at a driving voltage of 4000 V.

The mechanical resonance frequency was determined by performing multiple DIC measurements for different switching frequencies of the DEAs. A maximum deflection was observed at a switching frequency of 5.0 Hz. Figure 14 shows the frequency dependence of the fish fin deflection in the frequency range between 1 Hz and 7 Hz.

**Figure 15:**
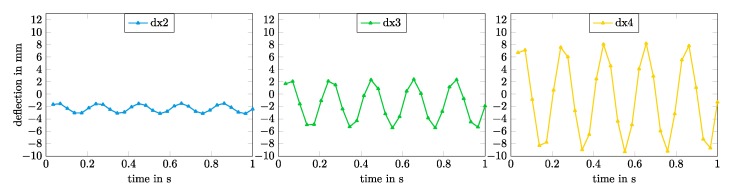
Measured time course for the deflections dx2 (**left**), dx3 (**middle**) and dx4 (**right**) at a 5.0 Hz switching frequency at a voltage of 4000 V.

The detected mechanical resonance frequency was expectedly lower than the predicted resonance frequency calculated from the electromechanical network model. The predicted value lied 1.385 Hz above the measured resonance frequency of 5.0 Hz. Figure 15 shows the detailed time courses for the single deflections dx2, dx3, and dx4 at a resonance frequency of 5.0 Hz.

A maximum deflection of 9.3 mm was measured at speckle point P4 at resonance frequency. In this case, a higher deflection to the left (negative) side was observed. This could be caused by several reasons. First of all, the alignment of the textile material might possibly be not absolutely straight along the length of the robot, leading to an unbalanced mechanical behavior. Furthermore, it could be assumed that the electrodes of the two DEAs were not absolutely identical. The manual application inherited some process tolerances that could also lead to a deviating electromechanical behavior of the DEAs. Another influence might be caused by not perfectly timed electrical signals of the DEAs. The used high voltage source did not provide a perfect phase control of the different switched channels between each other, which could lead to an overlap between the switching signals of the two DEAs. All of these possible influences on the accuracy could be reduced by automating the production process, including industrial production methods.

### 3.2. Movement in Water

The results of the investigations described so far considered the operation of the fish fin in air. Besides the operation mode in air, it was also of interest that the fish fin could be operated in water. For this, the outer electrodes on the left and right side of the robot could be contacted using the electrical admittance of the water itself. The electrical concept was therefore modified in such a way that the outer electrodes served as the common ground electrode, and the inner electrodes on both sides were contacted to the high voltage conducts. This concept allowed eliminating at least one wired connection to the robot. The robotic setup only needed two wired connections for the high voltage electrodes to perform its movement in water. In our preliminary test setups, we were able to prove the basic functionality. Therefore, the robot was clamped at its front side and held in a defined position under water. The waving motion of the fish fin was initiated by switching the high voltage electrodes for both DEAs consecutively. In this setup, the desired motion under water was proven (see Video S1 in the Supplementary Materials). A full characterization of the robot’s movement in water will be performed in our future work. The first results showed that the resonance frequency was significantly lower in water than in air. Furthermore, the steering of the robot and the exact control of its movement are challenges that will be solved in our future developments.

## 4. Conclusions

We successfully demonstrated a biomimetic fish fin-like robotic concept of a bending structure consisting of textile-elastomer compounds that was driven with DEAs on both sides. The robot was characterized in air by evaluating its deflection properties in a hanging setup. For this, DEAs on both outer sides of the fish fin were switched consecutively with a square-wave voltage signal of 4000 V. The simulation of the frequency-dependent behavior of the fish fin structure was done in two ways. The mechanical behavior of the 3D structure was calculated by using a finite element model that simulated the electromechanical coupling between the DEAs and the structure itself. This allowed us to consider that the structure would show the exact desired bending behavior with reasonable deflections. Figure 9 shows the outcome of the simulation for a static case without any dynamic frequency behavior. The results of the simulation showed that in a static case with an applied voltage of 5000 V, deflections of up to 10 mm at the end of the fin could be reached. The dynamic behavior of the fish fin under cyclic activation was modeled with an electromechanical network model, using analogies between mechanical characteristics and electrical network components. The dynamic case of consecutively switching the DEAs on both outer sides of the fish fin was considered in the electromechanical network model. The result of this investigation was that for dynamic switching, a maximum deflection could be expected at the calculated mechanical resonance frequency of 6.385 Hz (Figure 11). As mentioned before, the used network model overestimated the resonance frequency. The performed measurements underpinned this assumption. The measured resonance frequency in a hanging setup in air was detected at 5.0 Hz (Figure 14), which was 1.385 Hz below the calculated resonance frequency. This constituted a deviation of 27.7%. At resonance frequency, the maximum deflection of 9.3 mm was detected. Additionally, a basic functionality of the robotic concept to act in water was proven. In future developments, the presented bending behavior will serve as a propulsion and steering mechanism to build a more autonomous, self-driving robot. For that case the actuation in water is aspired.

## Supplementary Materials

The following are available at https://www.mdpi.com/2072-666X/11/3/298/s1, Video S1: Robot’s movement under water in a clamped setup.

## Abbreviations

The following abbreviations are used in this manuscript:

ABS	Acrylonitrile butadiene styrene
CF-UD	Carbon fiber unidirectional tape
DE	Dielectric elastomer
DEA	Dielectric elastomer actuator
DIC	Digital image correlation
EAP	Electroactive polymer
SBR	Styrene butadiene rubber
